# Comparative effects of SGLT2 inhibitors and GLP-1 receptor agonists on osteoarthritis risk in patients with type 2 diabetes mellitus: A multi-institutional cohort study

**DOI:** 10.1371/journal.pone.0353956

**Published:** 2026-07-20

**Authors:** Wen-Yu Jao, Chih‐Cheng Lai, Chi-Sheng Chien, Wei-Ting Lin

**Affiliations:** 1 Department of Orthopaedics, Chi Mei Medical Centre, Tainan, Taiwan; 2 Department of Internal Medicine, Chi Mei Medical Centre, Tainan, Taiwan; 3 Department of Orthopaedics, Chi Mei Medical Centre, Tainan, Taiwan; The Chinese University of Hong Kong, HONG KONG

## Abstract

**Objectives:**

Sodium-glucose co-transporter-2 (SGLT2) inhibitors and glucagon-like peptide-1 receptor agonists (GLP-1 RAs) have demonstrated metabolic and anti-inflammatory benefits, which may influence the risk of osteoarthritis (OA) in patients with type 2 diabetes mellitus (T2DM). However, their comparative impact on OA incidence remains unclear. This study aimed to evaluate and compare the effects of SGLT2 inhibitors and GLP-1 RAs on the risk of developing OA in patients with T2DM.

**Methods:**

A retrospective cohort study was conducted using the TriNetX database, including adults with T2DM who were newly prescribed either SGLT2 inhibitors or GLP-1 RAs between January 2017 and December 2019. After propensity-score matching, 452,445 patients were included in each group. The primary outcome was OA incidence over five years. Secondary outcomes included knee OA, hip OA, total knee arthroplasty (TKA), total hip arthroplasty (THA), and major joint injections.

**Results:**

The SGLT2 inhibitor group had a significantly lower risk of OA compared with the GLP-1 RAs group (HR, 0.921; 95% CI, 0.895–0.946), with a greater reduction observed for knee OA (HR, 0.831; 95% CI, 0.783–0.882). No significant differences were observed in THA or TKA risk. SGLT2 inhibitor users also had fewer major joint injections (HR, 0.915; 95% CI, 0.882–0.951).

**Conclusions:**

Among patients with T2DM, SGLT2 inhibitors were associated with a lower risk of OA, particularly knee OA and joint injections, compared to GLP-1 RAs. These findings support further investigation into the potential association between SGLT2 inhibitor use and lower OA-related outcomes.

## Introduction

Osteoarthritis (OA) is a common degenerative joint disease for which long-term treatment options are largely limited to surgical interventions. Several risk factors contribute to its development, including advancing age, obesity, trauma and type 2 diabetes mellitus (T2DM) [1,2]. Among these, T2DM is particularly notable, as it not only contributes to OA progression through mechanisms such as chronic low-grade inflammation, insulin resistance, and metabolic dysfunction but is also associated with a higher prevalence and severity of OA [3,4]. Individuals with T2DM are more likely to develop OA, experience accelerated disease progression, and face worse clinical outcomes compared to those without T2DM [4]. This highlights the pressing need for effective interventions targeting both metabolic and inflammatory pathways to reduce the incidence of OA, particularly in populations with T2DM.

Novel anti-diabetes medications, particularly sodium-glucose cotransporter 2 (SGLT2) inhibitors and glucagon-like peptide-1 receptor agonists (GLP-1 RAs), have revolutionized the treatment of T2DM. Beyond their primary role in glycemic control, these medications demonstrate pleiotropic effects by reducing cardio-renal-metabolic adverse outcomes [5]. Recent evidence suggests they may also address the shared metabolic and inflammatory pathways underlying both T2DM and OA [6–9]. These therapeutic agents show promise through multiple mechanisms: improving glycemic control, facilitating weight loss, and reducing systemic inflammation—all key factors in OA development. SGLT2 inhibitors lower glucose reabsorption and improve metabolic profiles, thereby reducing inflammation and promoting weight reduction [7,9,10]. These effects may help slow cartilage degradation and decrease the risk of OA onset. Similarly, GLP-1 RAs induce substantial weight loss and exert anti-inflammatory effects, targeting the metabolic and inflammatory pathways common to both T2DM and OA. Clinical studies of patients with T2DM have demonstrated that GLP-1 RA therapy is associated with significant benefits in OA management, including reduced consumption of symptom-relieving medications, slower cartilage loss in the medial femorotibial joint among patients with knee OA, and lower risk of both knee OA development and total knee replacement [11,12]. Despite their shared metabolic and anti-inflammatory effects, whether SGLT2 inhibitors and GLP-1 RAs are differentially associated with incident OA remains unclear. Therefore, this study aimed to compare the association of SGLT2 inhibitor versus GLP-1 RA initiation with subsequent OA-related outcomes in patients with T2DM. The findings may provide real-world comparative evidence to inform future research on metabolic therapies and musculoskeletal outcomes in patients with T2DM.

## Methods

### Database and study design

This cohort study used data from the TriNetX [13] database, which collects deidentified, patient-level data from electronic health records. Information in the TriNetX database comes from health care organizations (HCOs), typically academic health care centers, that collect data from their main and satellite hospitals and outpatient clinics. Available data include demographics, diagnoses (based on International Classification of Diseases, Tenth Revision, Clinical Modification codes), procedures (classified by International Classification of Diseases, Tenth Revision Procedure Coding System or Current Procedural Terminology), medications (Veterans Affairs Drug Classification System and RxNorm codes), laboratory tests (organized using Logical Observation Identifiers Names and Codes), and health care utilization records. We used the US Collaborative Network in TriNetX, which includes data from more than 100 million patients from 67 HCOs in the database. This study was conducted in accordance with the ethical principles outlined in the Declaration of Helsinki and was reviewed and approved by the Institutional Review Board of Chi Mei Medical Center (IRB No. 11402-E02). The requirement for informed consent was waived because this study used de-identified data.

### Patient selection

This study utilized the TriNetX Research Network database to identify the study population between January 1, 2017, and December 31, 2019.

Adult patients (aged ≥18 years) with T2DM who were using either SGLT2 inhibitors or GLP-1 RAs were identified using the 10th edition of the International Classification of Diseases (ICD-10) and RxNorm Concept Unique Identifiers. The diagnosis of T2DM was based on the ICD-10 code E11.

Patients with a prior diagnosis of osteoarthritis (ICD-10: M15–M19) were excluded from the study. To differentiate the two cohorts, one group was defined by the use of SGLT2 inhibitors and the other by the use of GLP-1 RAs. Patients who had a history of using the comparator medication were excluded from each respective cohort (e.g., individuals in the SGLT2 inhibitor cohort with prior GLP-1 RA use were excluded).

Only adults newly prescribed either an SGLT2 inhibitor or a GLP-1 RA were included. New users were defined as individuals with no prescriptions for the index medication or its comparator within the 12 months preceding the index date (defined as the date of the first prescription).

SGLT2 inhibitor use was identified based on prescriptions for canagliflozin (RxNorm: 1373458), dapagliflozin (1488564), empagliflozin (1545653), ertugliflozin (1992672), bexagliflozin (1617044), or sotagliflozin (2638675). GLP-1 RA use was identified through prescriptions for semaglutide (1991302), albiglutide (1534763), dulaglutide (1551291), liraglutide (4755968), lixisenatide (1440051), or exenatide (60548).

Patients were classified and followed according to their initially prescribed index medication, regardless of subsequent treatment discontinuation, switching, or add-on use of the comparator medication. Post-index treatment changes were not used to reclassify exposure status in the primary analysis.

### Covariates

We extracted covariates from each patient for the propensity score models, selecting variables that were likely to confound the relationship between the choice of SGLT2 inhibitors versus GLP-1 RAs and the prespecified outcomes [14]. These covariates included demographic factors, hemoglobin A1c levels, and comorbid conditions such as essential hypertension, ischemic heart disease, chronic kidney disease, cerebrovascular disease, and peripheral vascular disease, obesity, and concurrent medications (including metformin and insulin). A comprehensive list of covariates is provided in Table 1.

**Table 1:** Baseline characteristics before and after propensity-score matching. Continuous variables are presented as mean ± standard deviation and categorical variables as number (%). SMD, standardized mean difference. Covariate balance was primarily assessed using SMDs, with an SMD < 0.1 considered acceptable.

	Before matching				After matching			
	SGLT2 inhibitors	GLP-1 RAs	SMDs	P value	SGLT2 inhibitors	GLP-1 RAs	SMDs	P value
	(n = 586,195)	(n = 545,583)			(n = 452,445)	(n = 452,445)		
Age at Index								
Mean ± SD	60.9 ± 13.3	55.4 ± 14.1	0.402	< 0.001	58.3 ± 13.0	58.4 ± 12.7	0.009	0.242
Gender, n (%)								
Male	339,906 (57.9)	251,209(46.1)	0.238	< 0.001	236,950(52.4)	234,829 (51.9)	0.010	<0.001
Female	231,199 (39.4)	278,786(51.1)	0.237	< 0.001	203,322(44.9)	204,979 (45.3)	0.008	0.002
Race, n (%)								
White	333,785 (56.9)	311,503(57.1)	0.004	0.096	258,286(57.1)	258,962 (57.2)	0.002	0.151
Black	91,111(15.5)	94,780(17.4)	0.051	< 0.001	74,988(16.6)	74,915 (16.6)	0.001	0.836
Asian	33,635(5.7)	20,914(3.8)	0.089	< 0.001	19,419(4.3)	19,586 (4.3)	0.001	0.387
Hispanic	61,640(10.5)	55,464(10.1)	0.013	< 0.001	47,367(10.5)	46,743 (10.3)	0.007	0.031
Lab data								
HbA1c (%)	7.49 ± 2.03	7.33 ± 2.03	0.079	< 0.001	7.52 ± 2.08	7.43 ± 2.01	0.044	< 0.001
Concurrent medications, n (%)								
Metformin	387,000 (66.1)	320,342(58.7)	0.153	< 0.001	293,133(64.8)	290,658 (64.2)	0.013	0.112
Insulin	164,471 (28.1)	185,360(33.9)	0.126	< 0.001	126,034(27.9)	130,197 (28.8)	0.020	0.087
Comorbidities, n (%)								
Essential hypertension	97,418(16.6)	95,439(17.5)	0.024	< 0.001	76,361(16.9)	76,850 (16.9)	0.001	0.171
Ischemic heart disease	28,881(4.9)	17,588(3.2)	0.086	< 0.001	16,750(3.7)	16,928 (3.7)	0.001	0.322
Chronic kidney disease	14,220(2.4)	10,915(2.0)	0.027	<0.001	9,450(2.0)	9,799(2.1)	0.007	0.004
Cerebrovascular disease	8,894(1.5)	6,260(1.1)	0.035	<0.001	5,379(1.2)	5,723(1.2)	0.001	0.001
Peripheral vascular disease	6,932(1.2)	7,223(1.3)	0.009	< 0.001	5,290(1.2)	5,634(1.2)	0.001	0.001
Obesity	19,255(3.3)	29,879(5.5)	0.107	<0.001	17,388(3.8)	17,871(3.9)	0.005	0.215

### Outcome

Over the five-year follow-up period, the primary outcome was the incidence of OA, defined as the presence of ICD-10 codes M15–M19 recorded on at least two separate clinical encounters. This approach was used to improve diagnostic specificity and reduce potential misclassification, consistent with methods commonly adopted in claims-based epidemiological research. The incidence of OA was subsequently compared between patients prescribed SGLT2 inhibitors and those prescribed GLP-1 RAs.

To further investigate specific manifestations of OA and related interventions, several secondary outcomes were evaluated. These included the incidence of hip OA (ICD-10: M16), knee OA (ICD-10: M17), total hip arthroplasty (THA; CPT: 27130), total knee arthroplasty (TKA; CPT: 27447), and major joint injections (CPT: 20610, 20611). Hip OA and knee OA were defined as the corresponding ICD-10 diagnosis recorded on at least two separate clinical encounters during follow-up. Major joint injections were defined as at least two recorded injection events during the follow-up period to enhance diagnostic specificity and better reflect clinically relevant treatment exposure.

The rates of these outcomes were calculated and compared across the matched cohorts of patients receiving either SGLT2 inhibitors or GLP-1 RAs. Follow-up continued until the earliest of the following events: occurrence of the outcome, death, or December 31, 2024. The diagnostic, medication, exclusion, outcome, demographic, and laboratory data sources used to define the study cohorts, exposures, outcomes, and baseline covariates are summarized in S1 Table.

### Statistical analysis

Continuous variables were expressed as mean ± standard deviation (SD), while categorical variables were presented as counts and percentages. To minimize the impact of confounding factors, propensity scores were estimated using a logistic regression model including age at index, sex, race, HbA1c, obesity, essential hypertension, ischemic heart disease, chronic kidney disease, cerebrovascular disease, peripheral vascular disease, metformin use, and insulin use. Patients were matched in a 1:1 ratio using the TriNetX built-in greedy nearest-neighbor algorithm without replacement, with a caliper width of 0.2 standard deviations of the logit of the propensity score. Missing or unavailable covariate information was handled within the TriNetX built-in propensity-score matching workflow, and no additional imputation was performed by the investigators. Covariate balance was assessed using standardized mean differences, with an SMD < 0.1 considered acceptable. Hazard ratios (HRs) with corresponding 95% confidence intervals (CIs) were calculated for both primary and secondary outcomes using Cox proportional hazards models. Additionally, Kaplan–Meier analysis was used to evaluate time-to-event and frequency outcomes between groups. All statistical analyses were conducted using the TriNetX platform. Statistical significance was defined as P < 0.05.

## Results

### Patient selection

**Fig 1:**
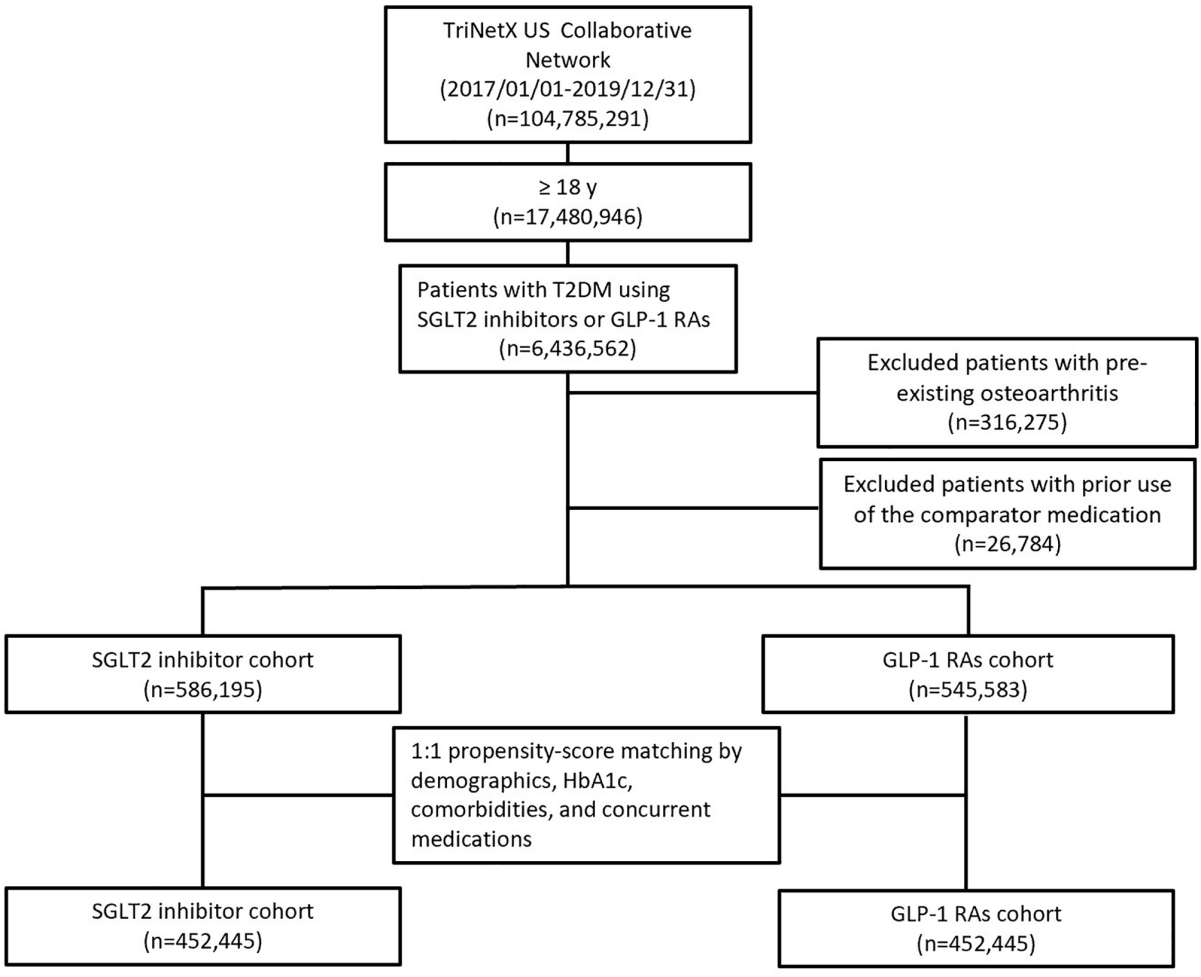
Flowchart of cohort construction. Patients with type 2 diabetes mellitus who initiated either sodium–glucose cotransporter 2 (SGLT2) inhibitors or glucagon-like peptide-1 receptor agonists (GLP-1 RAs) between January 1, 2017, and December 31, 2019, were identified from the TriNetX US Collaborative Network. Patients with pre-existing osteoarthritis or prior use of the comparator medication were excluded. The remaining patients were further restricted to new users of the index medication, defined as having no recorded use of the assigned drug class or comparator medication within the 12 months before the index date. After 1:1 propensity-score matching using baseline covariates, 452,445 patients were included in each group. T2DM, type 2 diabetes mellitus; SGLT2, sodium–glucose cotransporter 2; GLP-1 RAs, glucagon-like peptide-1 receptor agonists.

A flowchart of the cohort construction is provided in Fig 1. This study analyzed data from the TriNetX network (as of December 30, 2024), which includes 67 HCOs and 104,785,291 individuals. We selected patients aged 18 or older who had at least two HCO visits between 2017 and 2019, resulting in 17,480,946 eligible individuals. Among them, 6,436,562 patients had used either SGLT2 inhibitors or GLP-1 RAs. After applying exclusion criteria, the SGLT2 inhibitor cohort included 586,195 users, while the GLP-1 RAs cohort comprised 545,583. Using 1:1 PSM, we established a final cohort of 452,445 patients in each group.

### Demographic features of included patients

Table 1 presents a comparison between the SGLT2 inhibitor and GLP-1 RAs groups before and after PSM. Before matching, the SGLT2 inhibitor group was older on average than the GLP-1 RAs (60.9 ± 13.3 vs. 55.4 ± 14.1, P < 0.001). There were also significant differences in gender distribution, with a higher proportion of males in the SGLT2 inhibitor group (57.9% vs. 46.1%, P < 0.001). Racial composition differed significantly between groups, except for the proportion of White individuals (P = 0.096).

Regarding laboratory data, the SGLT2 inhibitor group had a slightly higher HbA1c level than the GLP-1 RAs group (7.49 ± 2.03 vs. 7.33 ± 2.03, P < 0.001). In terms of comorbidities, the SGLT2 inhibitor group had a lower prevalence of essential hypertension, ischemic heart disease, chronic kidney disease, cerebrovascular disease, and peripheral vascular disease than the GLP-1 RAs group (P < 0.001 for all).

After PSM, 452,445 matched patients were included in each group, leading to a more balanced distribution of baseline characteristics. All standardized mean differences after matching were below 0.1, indicating acceptable covariate balance. Although chronic kidney disease (P = 0.004), cerebrovascular disease (P = 0.001), peripheral vascular disease (P = 0.001), and HbA1c levels (P < 0.001) remained statistically different between groups, the magnitude of these differences was small according to SMD values.

## Primary outcome

After a five-year follow-up, patients with T2DM in the SGLT2 inhibitor group had a significantly lower risk of osteoarthritis (HR, 0.921; 95% CI, 0.895–0.946; P < 0.001) compared to those in the GLP-1 RAs group (Table 2).

**Table 2:** Comparative risk assessment of SGLT2 inhibitors and GLP-1 RAs. Data are presented as number (%). Hazard ratios were calculated using Cox proportional hazards models. OA, osteoarthritis; THA, total hip arthroplasty; TKA, total knee arthroplasty; CI, confidence interval. Statistical significance was defined as P < 0.05.

	SGLT2 inhibitors (n = 452,445)	GLP-1 RAs (n = 452,445)	Hazard ratio	95% CI	P value
OA	9,259 (2.05)	10,575 (2.34)	0.921	0.895-0.946	<0.001
Hip OA	757 (0.17)	856 (0.19)	0.931	0.843-1.025	0.111
Knee OA	1,927 (0.43)	2,438 (0.54)	0.831	0.783-0.882	<0.001
Major joint injection	5,163 (1.14)	5,936 (1.31)	0.915	0.882-0.951	<0.001
THA	90 (0.02)	81 (0.02)	1.168	0.865-1.577	0.311
TKA	79 (0.02)	95 (0.02)	0.877	0.651-1.182	0.389

## Secondary outcome

**Fig 2:**
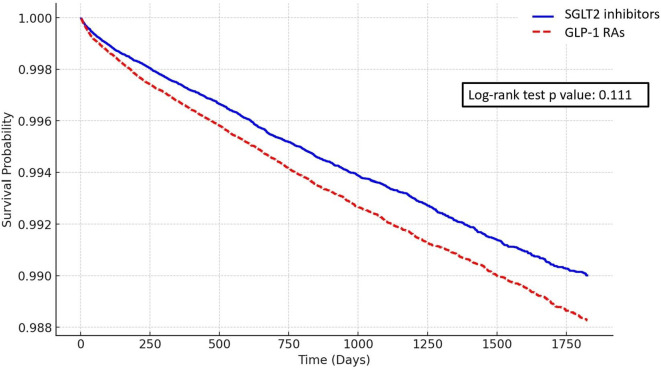
Comparison of hip osteoarthritis occurrence with a 5-year follow-up. Kaplan–Meier curve comparing hip osteoarthritis occurrence between the SGLT2 inhibitor and GLP-1 RA groups during the 5-year follow-up period. The log-rank test showed no statistically significant difference between groups. SGLT2, sodium–glucose cotransporter 2; GLP-1 RA, glucagon-like peptide-1 receptor agonist.

**Fig 3:**
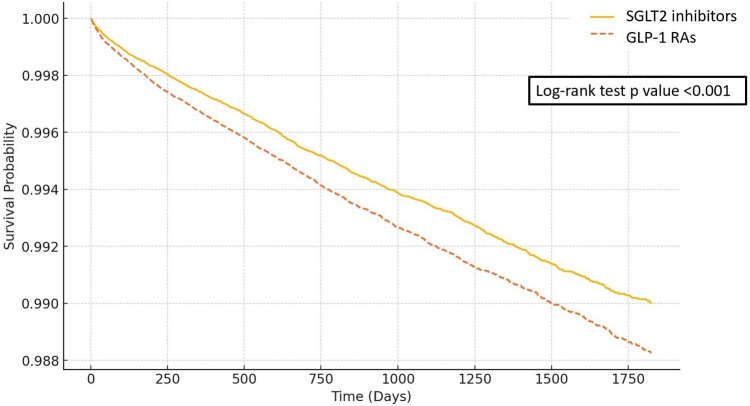
Comparison of knee osteoarthritis occurrence with a 5-year follow-up. Kaplan–Meier curve comparing knee osteoarthritis occurrence between the SGLT2 inhibitor and GLP-1 RA groups during the 5-year follow-up period. The SGLT2 inhibitor group showed a significantly lower risk of knee osteoarthritis than the GLP-1 RA group. SGLT2, sodium–glucose cotransporter 2; GLP-1 RA, glucagon-like peptide-1 receptor agonist.

When analyzing the incidence of hip and knee osteoarthritis separately, the SGLT2 inhibitor group exhibited a slightly lower occurrence of hip osteoarthritis (HR, 0.931; 95% CI, 0.843–1.025); however, survival analysis indicated that this difference was not statistically significant (log-rank test, P = 0.111). In contrast, the SGLT2 inhibitor group had a significantly lower risk of knee osteoarthritis compared to the GLP-1 RAs group (HR, 0.831; 95% CI, 0.783–0.882), with survival analysis confirming statistical significance (log-rank test, P < 0.001). Kaplan–Meier survival plots were provided in Figs 2–3.

**Fig 4:**
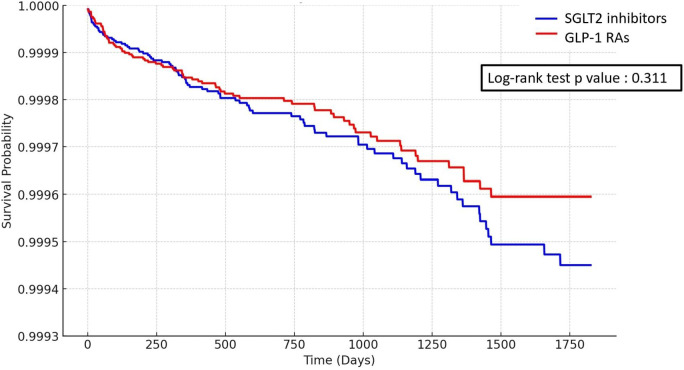
Comparison of total hip arthroplasty occurrence with a 5-year follow-up. Kaplan–Meier curve comparing total hip arthroplasty occurrence between the SGLT2 inhibitor and GLP-1 RA groups during the 5-year follow-up period. No statistically significant difference was observed between groups. SGLT2, sodium–glucose cotransporter 2; GLP-1 RA, glucagon-like peptide-1 receptor agonist; THA, total hip arthroplasty.

**Fig 5:**
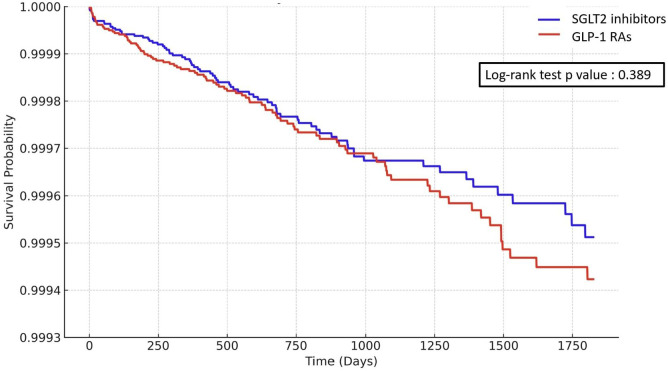
Comparison of total knee arthroplasty occurrence with a 5-year follow-up. Kaplan–Meier curve comparing total knee arthroplasty occurrence between the SGLT2 inhibitor and GLP-1 RA groups during the 5-year follow-up period. No statistically significant difference was observed between groups. SGLT2, sodium–glucose cotransporter 2; GLP-1 RA, glucagon-like peptide-1 receptor agonist; TKA, total knee arthroplasty.

**Fig 6:**
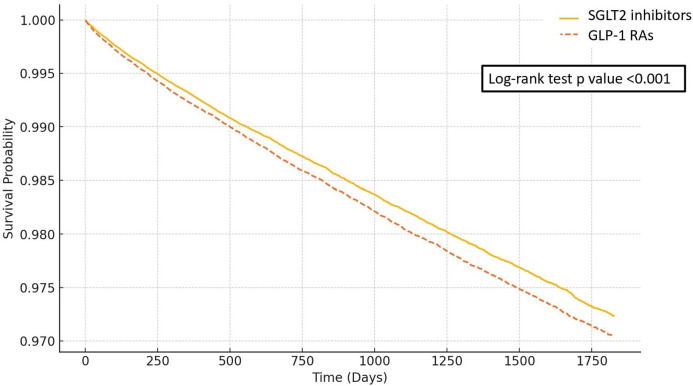
Comparison of major joint injection occurrence with a 5-year follow-up. Kaplan–Meier curve comparing major joint injection occurrence between the SGLT2 inhibitor and GLP-1 RA groups during the 5-year follow-up period. The SGLT2 inhibitor group showed a significantly lower occurrence of major joint injection than the GLP-1 RA group. SGLT2, sodium–glucose cotransporter 2; GLP-1 RA, glucagon-like peptide-1 receptor agonist.

There were no significant differences between the two groups in the incidence of THA (HR, 1.168; 95% CI, 0.865–1.577; P = 0.311) or TKA (HR, 0.877; 95% CI, 0.651–1.182; P = 0.389). However, the SGLT2 inhibitor group had a lower rate of major joint injections compared to the GLP-1 RAs group (1.14% vs. 1.31%; HR, 0.915; 95% CI, 0.882–0.951; P < 0.001) (Table 2). Survival analysis indicated that this difference was statistically significant (log-rank test, P < 0.001). Kaplan–Meier survival plots were also provided in Figs 4–6.

## Discussion

This study provides real-world comparative evidence regarding the effects of SGLT2 inhibitors and GLP-1 RAs on the incidence of OA in patients with T2DM. Through propensity-score matching and analysis of a large TriNetX cohort, we examined the association between these therapeutic agents and OA-related outcomes while reducing measured baseline confounding.

The results indicate that the use of SGLT2 inhibitors was associated with a lower incidence of OA compared to GLP-1 RAs. Specifically, the SGLT2 inhibitor cohort exhibited a significantly lower overall OA risk (HR: 0.921, 95% CI: 0.895–0.946). This association was primarily observed for knee OA (HR: 0.831, 95% CI: 0.783–0.882), whereas the difference in hip OA was directionally lower but not statistically significant (HR: 0.931, 95% CI: 0.843–1.025). Additionally, the SGLT2 inhibitor cohort demonstrated a lower likelihood of requiring major joint injections (HR: 0.915, 95% CI: 0.882–0.951), suggesting a lower observed frequency of OA-related procedural treatment in this cohort.

SGLT2 inhibitors have demonstrated benefits in patients with diabetes, and their anti-inflammatory properties may provide biological plausibility for the observed association with OA-related outcomes. Recent studies [9,15–17] indicate SGLT2 inhibitors offer therapeutic benefits beyond glucose control, through anti-inflammatory effects. Agents such as empagliflozin, dapagliflozin, and canagliflozin have been shown to reduce chronic low-grade inflammation by modulating macrophage activity [18,19]. Rykova et al. [15] indicate SGLT2 inhibitors promote macrophage polarization from the pro-inflammatory M1 to the anti-inflammatory M2 phenotype, lowering cytokines such as tumor necrosis factor-alpha (TNF-α), interleukin-1 beta (IL-1β), interleukin-6 (IL-6), and C-reactive protein (CRP). They also suppress key inflammatory signaling pathways, including nuclear factor kappa B (NF-κB), Toll-like receptor 4 (TLR4), AMP-activated protein kinase (AMPK), and the Janus kinase/signal transducer and activator of transcription (JAK/STAT) pathway. These anti-inflammatory mechanisms provide biological plausibility for the observed association; however, the present study did not directly assess inflammatory biomarkers, cartilage structure, radiographic progression, or patient-reported symptoms. Therefore, mechanistic interpretations should remain hypothesis-generating.

GLP-1 RAs also offer advantages, particularly in glycemic control and weight reduction. Zhu et al. [11] reported improvements in OA-related symptoms and a reduction in the use of symptom-control medications in patients treated with GLP-1 RAs. Recent clinical and preclinical evidence suggests that GLP-1-RAs may have a dual role in OA management beyond their metabolic effects [20–23]. A real-world cohort study by Porto et al. [20] found that in obese patients with hip or knee OA, GLP-1-RAs use significantly reduced the risk of total joint arthroplasty, suggesting a potential association with OA-related clinical outcomes. Meurot et al. [22] demonstrated that GLP-1 RAs and its analogues exert anti-inflammatory and anti-catabolic effects on joint tissues, suggesting potential biological relevance to OA-related pathways.

Importantly, this study represents the first head-to-head comparison between SGLT2 inhibitors and GLP-1 RAs in evaluating OA incidence. By utilizing a large-scale national database, our findings offer meaningful insights into the potential differential impact of these therapeutic agents on osteoarthritis, highlighting the importance and novelty of this research.

### Clinical and research implications

These findings may be considered hypothesis-generating and may inform future studies evaluating whether antidiabetic drug selection is associated with musculoskeletal outcomes in patients with T2DM. They also underscore the need for individualized treatment strategies, as different therapeutic agents may be associated with different musculoskeletal outcome profiles depending on patient-specific risk factors and comorbidities.

From a research perspective, these findings warrant further investigation into the mechanisms that may explain the observed association between SGLT2 inhibitor use and lower OA-related outcomes. Future studies could explore the effects of these agents on cartilage tissue, inflammatory cytokine profiles, oxidative stress markers, and other pathways relevant to OA development. Additionally, long-term prospective studies and randomized controlled trials are needed to confirm the observed associations and to determine whether SGLT2 inhibitors influence OA onset or progression in broader patient populations.

### Limitations and strengths

This study has several limitations. First, its retrospective observational design precludes the establishment of causality. Second, although propensity-score matching was used to improve baseline comparability, residual confounding cannot be excluded. Unmeasured or incompletely captured factors, including changes in body weight, duration and severity of diabetes, physical activity, healthcare utilization intensity, medication adherence, and post-index treatment changes, may have influenced the observed associations. Patient-level post-index medication trajectories could not be reliably extracted within the aggregate TriNetX analytic workflow available for this study. Therefore, the proportion of patients who subsequently discontinued, switched to, or combined the comparator medication during follow-up could not be directly ascertained, and a sensitivity analysis using an as-treated framework was not feasible. As patients were classified according to their initially prescribed index medication in the primary analysis, potential exposure misclassification related to post-index treatment discontinuation, switching, or add-on use cannot be fully excluded. Third, OA-related outcomes were identified using ICD-10 diagnosis codes and procedure codes rather than radiographic findings, clinical severity grading, or patient-reported symptoms. Therefore, outcome misclassification may have occurred, and this study could not distinguish different stages or severities of OA.

This study also has several strengths. The use of a large multi-institutional database allowed the inclusion of a substantial number of patients with T2DM and enabled evaluation of multiple OA-related outcomes. In addition, the active-comparator, new-user design may have reduced confounding related to treatment indication and prior medication exposure. Propensity-score matching further improved measured baseline comparability between the SGLT2 inhibitor and GLP-1 RAs groups.

## Conclusion

This study demonstrates that SGLT2 inhibitor use is associated with a lower risk of OA compared to GLP-1 RA use in patients with T2DM, particularly for knee OA and major joint injections. These findings suggest that antidiabetic drug selection may be relevant to OA-related outcomes in this high-risk population and underscore the importance of considering both metabolic and musculoskeletal health in individualized treatment strategies. Given the retrospective observational design, these findings should be interpreted as hypothesis-generating and warrant further confirmation in prospective studies.

## Supporting information

S1 Table Code definitions and data sources used in this study. This table contains only diagnostic, medication, procedure, demographic, and laboratory code definitions used to construct the study cohorts and outcomes. It does not contain patient-level data or individual participant identifiers.
(DOCX)

S1 Checklist STROBE checklist. Completed STROBE checklist for this observational cohort study.
(DOCX)
