# Supplementary material for: Comparative effects of SGLT2 inhibitors and GLP-1 receptor agonists on osteoarthritis risk in patients with type 2 diabetes mellitus: A multi-institutional cohort study
Source: PLoS One. 2026 Jul 20;21(7):e0353956. doi: 10.1371/journal.pone.0353956 (PMC13384269; doi:10.1371/journal.pone.0353956)
Supplement: S1 Table — This table contains only diagnostic, medication, procedure, demographic, and laboratory code definitions used to construct the study cohorts and outcomes. It does not contain patient-level data or individual participant identifiers. (DOCX) [file pone.0353956.s001.docx]

S1 Table. Codes and data sources used to define study cohorts, exposures, outcomes, and baseline covariates

| **Category** | **Variable / Definition** | **Code(s) / Data Source** | **Notes** |
| --- | --- | --- | --- |
| Study population | Type 2 diabetes mellitus | ICD-10: E11 | Used to identify patients with T2DM |
| Exclusion criterion | Pre-existing osteoarthritis | ICD-10: M15–M19 | Patients with prior OA diagnosis before the index date were excluded |
| Exposure | SGLT2 inhibitor use | RxNorm: canagliflozin 1373458; dapagliflozin 1488564; empagliflozin 1545653; ertugliflozin 1992672; bexagliflozin 1617044; sotagliflozin 2638675 | Index exposure group; patients with prior GLP-1 RA use were excluded |
| Exposure | GLP-1 RA use | RxNorm: semaglutide 1991302; albiglutide 1534763; dulaglutide 1551291; liraglutide 4755968; lixisenatide 1440051; exenatide 60548 | Comparator exposure group; patients with prior SGLT2 inhibitor use were excluded |
| Primary outcome | Overall osteoarthritis | ICD-10: M15–M19 | Defined as OA diagnosis recorded on at least two separate clinical encounters during follow-up |
| Secondary outcome | Hip osteoarthritis | ICD-10: M16 | Defined as hip OA diagnosis recorded on at least two separate clinical encounters during follow-up |
| Secondary outcome | Knee osteoarthritis | ICD-10: M17 | Defined as knee OA diagnosis recorded on at least two separate clinical encounters during follow-up |
| Secondary outcome | Total hip arthroplasty | CPT: 27130 | Used to identify THA during follow-up |
| Secondary outcome | Total knee arthroplasty | CPT: 27447 | Used to identify TKA during follow-up |
| Secondary outcome | Major joint injection | CPT: 20610, 20611 | Defined as at least two recorded injection events during follow-up |
| Covariate | Essential hypertension | ICD-10: I10 | Primary hypertension |
| Covariate | Ischemic heart disease | ICD-10: I20–I25 | Includes angina pectoris, acute myocardial infarction, subsequent myocardial infarction, complications following myocardial infarction, and chronic ischemic heart disease |
| Covariate | Chronic kidney disease | ICD-10: N18.x | Includes CKD stages 1–5 and end-stage renal disease |
| Covariate | Cerebrovascular disease | ICD-10: I60–I69 | Includes nontraumatic intracranial hemorrhage, cerebral infarction, other cerebrovascular diseases, and sequelae of cerebrovascular disease |
| Covariate | Peripheral vascular disease | ICD-10: I70.2, I73.x, I77.x | Includes atherosclerosis of native arteries of the extremities, other peripheral vascular diseases, and selected arterial disorders |
| Covariate | Obesity | ICD-10: E66.x | Includes obesity and morbid obesity |
| Covariate | Insulin use | RxNorm medication records; ICD-10: Z79.4 | Medication exposure was identified using TriNetX medication records. Z79.4 indicates long-term current use of insulin |
| Covariate | Metformin use | RxNorm medication records; ICD-10: Z79.84 | Medication exposure was identified using TriNetX medication records. Z79.84 indicates long-term current use of oral hypoglycemic drugs and is not specific to metformin |
| Covariate | Race and ethnicity | Demographic data field | Derived from structured demographic data within TriNetX |
| Covariate | HbA1c | Laboratory data field | Captured using the TriNetX built-in laboratory data source |

This table contains only diagnostic, medication, procedure, demographic, and laboratory code definitions used to construct the study cohorts and outcomes. It does not contain patient-level data or individual participant identifiers.
